# Do social factors and country of origin contribute towards explaining a “Latina paradox” among immigrant women giving birth in Germany?

**DOI:** 10.1186/s12889-019-6523-9

**Published:** 2019-02-12

**Authors:** Kim Alexandra Zolitschka, Céline Miani, Jürgen Breckenkamp, Silke Brenne, Theda Borde, Matthias David, Oliver Razum

**Affiliations:** 1 Department of Epidemiology and International Public Health, School of Public Health, Bielefeld University, Bielefeld, Germany; 2 Department of Gynaecology, Campus Virchow-Klinikum, Charité-Universitätsmedizin Berlin, Berlin, Germany; 3 Alice Salomon Hochschule, Berlin, Germany

**Keywords:** Latina paradox, Pregnancy outcome, Turkey, Lebanon, Germany

## Abstract

**Background:**

The “Latina paradox” describes the unexpected association between immigrant status, which is often correlated to low socioeconomic status, and low prevalence of unfavourable birth outcomes. Social (e.g. culture, religion) and/or non-social factors related to country of origin are potentially responsible for this paradox.

**Methods:**

Questionnaire survey of 6413 women delivering in three large obstetric hospitals in Berlin (Germany) covering socioeconomic and migration status, country of origin (Turkey, Lebanon), and acculturation. Data was linked with routine obstetric data. Logistic regressions were performed to assess the effect of acculturation, affinity to religion and country of origin on preterm birth and small-for-gestational-age (SGA).

**Results:**

Immigrant women with a low level of acculturation (reference) were less likely to have a preterm birth than those who were highly acculturated (aOR: 1.62, 95%CI: 1.01–2.59), as were women from Turkey compared to non-immigrants (aOR: 0.49, 95%CI: 0.33–0.73). For SGA, we found no epidemiologic paradox; conversely, women from Lebanon had a higher chance (aOR: 1.72, 95%CI: 1.27–2.34) of SGA. Affinity to religion had no influence on birth outcomes.

**Conclusions:**

There is evidence that low acculturation (but not affinity to religion) contributes towards explaining the epidemiologic paradox with regard to preterm birth, emphasising the influence of socioeconomic characteristics on birth outcomes. The influence of Turkish origin on preterm birth and Lebanese origin on SGA suggests that non-social factors relating to the country of origin are also at play in explaining birth outcome differences, and that the direction of the effect varies depending on the country of origin and the outcome.

**Electronic supplementary material:**

The online version of this article (10.1186/s12889-019-6523-9) contains supplementary material, which is available to authorized users.

## Background

Immigrants tend to have a lower socio-economic status and poorer health than the majority population of the country they migrated to [1]. However, in the US, Hispanic women have been shown to benefit from a specific form of the “healthy migrant effect” and to have better birth outcomes than white women despite their lower socioeconomic status [2]. This phenomenon, often called the “Latina Paradox”, was found for preterm birth, low birth weights (LBW) and small-for-gestational-age (SGA) when comparing native and immigrant population groups [3–5]. So far, the “Latina Paradox” has mostly been framed as being associated with origin from a particular country (Mexico) or region (Latin America). Yet “country of origin” is a proxy for several concepts that need to be disentangled:

- A set of immutable factors related to the genetics on population level that we will call “non-social factors”. Non-social factors are not immediately affected by migration. These factors, which include maternal height and genetic polymorphisms among others on population level, stem from the country of origin. They probably vary across different countries of origin [4, 6].
- Social factors brought along from the country of origin such as culture and affinity to religion, which may change over time.
- Social position of immigrants in the target country of the migration, in particular the socioeconomic status of immigrants from a particular country or region relative to the host population – a confounder that needs to be controlled for when analysing the “Latina paradox” [4, 7].

Social factors include religion, culture, and related behaviours. Such factors vary between countries of origin [4, 6]. Immigrants bring along traditions, values and behaviour from their country of origin, which may positively (or negatively) affect perinatal outcomes [4, 8]. Such cultural factors, however, are likely to change in the course of time spent in the target country and over generations. They may be adapted to the culture of the new country, along with other health behaviours, which could influence birth outcomes [9].

Several studies have sought to explore a potential Latina Paradox in Europe, looking at the relationship between birth outcomes and country of origin. For example, in Belgium, Jacquemyn et al. (2012) and Racape et al. (2016) found that being of Turkish or North African origin had a protective influence on preterm birth and low birth weight, respectively. [10, 11]. In The Netherlands Schulpen et al. (2005) reported higher death rates for newborns of immigrant women (Antillan, Turkish and Moroccan) compared to the Dutch population [12]. In Sweden, a large birth register study including women of many different origins produced less clear results: women from Chile and Syria were the only non-Swedes who showed better results than the native population in terms of LBW and preterm birth, leading the authors to conclude that the healthy migrant effect is ethnic- and outcome-specific [13]. Finally, in Germany, a recent study by David et al. (2017), which used the same data set that we will analyse, showed a lower frequency of preterm birth among immigrant women [14]. However, similar to the other studies mentioned above, the authors did not investigate this phenomenon in detail.

To better disentangle the role of the different sets of influencing factors including the role of the cultural factors and country of origin in explaining the Latina paradox, we take two steps. First, we investigate whether a Latina paradox regarding two birth outcomes commonly examined in this context, preterm birth and SGA, can be observed in immigrant women stemming from countries or regions other than Mexico or Latin America (here Turkey, Lebanon, EU15, EU27, other Europe, Middle East (excluding Turkey and Lebanon), North Africa, Sub-Saharan Africa, Far East, Latin America and Caribbean and North America), and in a target country different from the US (here Germany). We then examine whether the effect differs between countries of origin after adjusting for socioeconomic status in the target country. If this is the case, non-social components may play a role in explaining the Latina paradox.

Second, we assess whether the Latina paradox is (at least partly) explained by social factors which non-immigrant women are not exposed to, e. g. religion and culture of the country of origin. Effects of social factors linked to the country of origin are more likely to be causally associated with the birth outcomes if they vary with degree of acculturation, after adjusting for socioeconomic status. If social factors play a role in the Latina paradox, evidence that non-social differences matter as well will become stronger when – after adjusting for socioeconomic status, acculturation and religion – differences in outcomes remain.

## Methods

### Setting

The study is set in Berlin, Germany. In 2014, 20.3% of Germany’s population (16.4 out of 80.9 million persons) and 25.3% of Berlin’s population (911,000 out of 3.7 million inhabitants) had a migration background. This term comprises persons who immigrated themselves as well as persons born in Germany who have at least one immigrant parent [15]. The largest immigrant group in Berlin originates from Turkey (220,000 persons) [16].

### Data sources

Data were collected between January 2011 and January 2012 at three large maternity hospitals in Berlin. Inclusion criteria were age 18 years and above, giving live birth at 24+ completed weeks of gestation and permanent German residency [17]. Women were interviewed by trained, female study staff with a standardised face-to-face interview and a questionnaire available in eight languages including Turkish, Arabic, English, and French. When necessary, an interpreter was consulted. Uniformly collected and standardised obstetric process and outcome data (the “perinatal data”) were linked from the hospital databases.

### Main outcome variables

We selected preterm births and SGA newborns as outcomes of interest as both have been used in previous studies exploring the Latina Paradox [4, 5]. Preterm births and SGA have an increased risk for perinatal morbidity, mortality and lifetime complications [18, 19]. Main risk factors for these two unfavourable birth outcomes include low social status [20, 21], low social support [22, 23], smoking [24, 25], absence of religious belief [26, 27], high or low maternal age [20, 28], low or high body mass index (BMI) [29, 30] and pre-existing maternal medical conditions [30, 31]. Overall, preterm birth and SGA incur high healthcare costs and are responsible for most infant deaths [32].

Preterm birth was defined as a birth which takes place before the end of the 37th week of pregnancy. SGA was defined as a birth weight below the defined limit for gestational age and sex (10th percentile). Adjusted data that served as reference dataset are available from the 23rd to the 43rd week of gestation for Germany [33].

### Determinant variables

*Immigrant status* of the women was defined based on their own and their parents’ country of birth [34]. Women were classified as 1st generation immigrants if they were born outside Germany and as 2nd generation women if they were born in Germany and both parents were born abroad. Women with both parents born in Germany served as reference group (non-immigrant women). Additional women with only one parent born abroad (*n* = 302) were grouped with the non-immigrant women (previous analyses had shown them to be quite similar to women with non-immigrant women) [35].

*Acculturation* was measured with items of the “Frankfurt Acculturation Scale” (FRAKK) [36]. The required information for the acculturation was obtained from 1st and 2nd generation women. Scores ranged from 13 to 90 on a scale from 0 to 90. A high score means a high acculturation. Acculturation was grouped in three equal-sized categories: low (13–38), medium (40–65) and high (65–90). Low acculturation served as reference group.

*Affinity to religion* was grouped as following: no religion, no affinity to religion, low affinity to religion, medium affinity to religion and high affinity to religion. No religion served as reference group. Reporting of an affinity to religion was found to be consistent with answers to another question regarding religion in the FRAKK questionnaire.

*Country of origin* was used to single out women from the two largest immigrant groups in the dataset, i.e. women from Turkey and Lebanon. Non-immigrant women served as reference group.

Besides *acculturation*, *affinity to religion* and the *country of origin* we consider the following covariates, which may influence birth outcomes:

- *Monthly household income* (categorised as < 900 EUR, 900–1500 EUR, 1500–2600 EUR and > 2600 EUR) and *education* (low (no degree/primary education), medium (lower secondary education) and high (upper secondary/high education)) reflect social status [31, 37]
- *Maternal age* (grouped as 18–24, 25–29, 30–34 and > 35 years), *diabetes mellitus* (recorded in antenatal card) and *preterm birth in anamnesis* (only for preterm birth) [20, 28]
- *Presence of family members in Berlin* (at least one vs. none) reflects social support [22, 23]
- *Smoking* (non-smoker vs. occasional smoker and smoker during pregnancy) [24, 25]

The smoking status defined through responses to our questionnaire was found to be consistent with the smoking-related variable in the routine perinatal data set. For the variable “smoking” values were missing for 288 women. Those data sets were excluded from the analysis. Missing data for other variables were imputed. Information on monthly household income was missing in 10.2% and on affinity in religion in 5.2%. Imputation procedures using the average of five iterations based on linear or polytomous regression analyses were conducted. The imputations were based on age, migrant status and education.

### Statistical analyses

Chi Square Tests were conducted to determine the relation of the different countries of origin to the outcomes. Separate logistic regressions were conducted to estimate Odds Ratios (OR) for the influence of acculturation, affinity to religion and country of origin on preterm and SGA births while controlling for potential confounders. Linear regression analyses were used to check for multicollinearity of confounders (data not shown as no statistical evidence for collinearity was detected). The dependent variables were the birth outcomes: preterm birth and SGA birth. Regression models were adjusted for the following confounders: smoking, maternal age, diabetes mellitus, family members in Berlin and possible predictors: education, monthly household income and migrant status. For the analysis of preterm birth, preterm birth in anamneses is an additional potential confounder. Adjusted odds ratios (aOR) were calculated with 95% confidence intervals (CI). The software “IBM SPSS Statistics 23” was used for the analysis. Statistical significance was defined as *p* < 0.05.

## Results

8157 women delivered in the three hospitals in the period of data collection. 7100 women participated in the study (response rate 89.6%) [17]. Complete data for all variables relevant for the analysis was available for 6413 out of the 7100 women. Of these women, 2552 were 1st generation immigrants (subgroups: 561 from Turkey and 317 from Lebanon); 885 were 2nd generation women. The proportion of women with low education was higher in the immigrant groups (1st generation: 25.0%; 2nd generation: 9.4%) than the non-immigrant group (3.3%), while the proportion of women with high income was lower in the immigrant groups (1st generation: 11.2%; 2nd generation: 10.6%) compared to non-immigrant women (37.4%). First generation women had a lower diabetes prevalence (0.6%) than non-immigrant women (1.1%), and diabetes prevalence was particularly low among women from Turkey (0.3%) and Lebanon (0.0%). The majority of non-immigrant women stated they followed no religion (49.6%) or had no or little affinity to religion (19.7%) whereas many immigrant women reported a high affinity to religion (1st generation: 41.1%; 2nd generation: 45.8%) (see Table 1). 14.3% of the 1st generation migrants had a low acculturation level vs. 2.6% of the 2nd generation women.

**Table 1:** Distribution of socio-demographic characteristics and obstetric indicators (in %, mean, SD) among study participants, by migration status, Berlin, Germany, 2011/12

		1st generation immigrants					
		total (all origins)	Turkish origin	Lebanese origin	2nd generation women	non-immigrant women	total
Study population (n)		2552	561	317	885	2976	6413
Maternal age	n=						
18–24 years	1288	19,9	20.0	19.6	18.3	20.7	20.1
25–29 years	1732	27.3	26.4	26.2	25.9	27.1	27.0
30–34 years	1883	29.5	28.5	27.4	30.6	28.9	29.4
35+ years	1510	23.3	25.1	26.8	25.2	23.3	23.5
Mean (SD)		29.9 (5.8)	29.3 (5.9)	29.5 (6.0)	30.0 (5.8)	30.0 (5.8)	29.8 (5.8)
Educational attainment	n=						
High	2521	34.3	41.0	39.4	16.8	50.2	39.3
Medium	3071	40.6	45.8	47.0	73.8	46.5	47.9
Low	821	25.1	13.2	13.6	9.4	3.3	12.8
Family members in Berlin	n=						
Yes	4299	53.0	67.6	61.5	92.8	28.6	67.0
No	2114	47.0	32.4	38.5	7.2	71.4	33.0
Affinity to religion	n=						
No religion	1777	10.0	8.8	8.1	5.4	49.6	27.7
No affinity to religion	313	3.5	3.5	1.9	2.7	6.7	4.9
Low affinity to religion	664	8.4	8.2	6.3	7.2	13.0	10.4
Medium affinity to religion	1901	37.1	36.6	30.6	38.9	20.5	29.6
High affinity to religion	1758	41.1	42.9	53.1	45.8	10.2	27.4
Smoking during pregnancy	n=						
No	5090	80.7	77.9	80.1	70.5	80.8	79.4
Yes	1323	19.3	22.1	19.9	29.5	19.2	20.6
Household income (monthly)	n=						
< 900 EUR	1572	19.8	20.8	13.8	24.7	28.5	24.5
900–1500 EUR	1228	27.1	29.3	34.4	22.6	11.3	19.1
1500–2600 EUR	2122	41.9	37.2	38.1	42.0	22.8	33.1
> 2600 EUR	1491	11.2	12.6	13.7	10.6	37.4	23.2
Diabetes mellitus	n=						
no	6353	99.4	99.6	99.7	98.6	98.9	99.1
yes	60	0.6	0.4	0.3	1.4	1.1	0.9
Preterm birth in anamneses	n=						
no	6191	96.8	97.9	95.3	96.5	96.3	96.5
yes	222	3.2	2.1	4.7	3.5	3.7	3.5
Acculturation	n=						
Low	387	14.3	6.1	10.0	2.6	0.0	6.0
Medium	2016	59.4	61.2	61.9	56.6	0.0	31.4
High	4010	26.3	32.7	28.1	40.8	100	62.5
Preterm birth	n=						
No	5786	90.8	94.7	91.8	91.2	89.4	90.2
Yes	627	9.2	5.3	8.2	8.8	10.6	9.8
SGA birth	n=						
No	5536	85.7	88.8	79.2	88.4	86.3	86.3
Yes	877	14.3	11.2	20.8	11.6	13.7	13.7

*SD* standard deviation

A table of countries of origin shows the distribution of 1st generation immigrants grouped in EU15, EU27, other Europe, Middle East (excluding Turkey and Lebanon), North Africa, Sub-Saharan Africa, Far East, Latin America and Caribbean and North America (see Additional file 1). Overall 9.8% of the births were preterm, with 10.6% in non-immigrant women. The lowest proportion of preterm births was in women from Turkey (5.3%) (see Table 1).

Additional file 2 shows the relation of the country of origin to the birth outcomes using Chi Square Tests. For preterm birth the regions, Sub-Saharan Africa, Latin America and Caribbean and the country Turkey were significant (see Additional file 2). Regarding SGA only Lebanon had a statistically significant association with the outcome.

Table 2 and Additional file 3 show the influence of the country of origin on preterm birth, while controlling for medical variables, age and smoking (model 1), socioeconomic variables (model 2), as well as affinity to religion and acculturation (model 3). In all models, the association between a Turkish origin and preterm birth was statistically significant (model 3, Turkish origin: aOR 0.49, 95%CI: 0.33–0.73). Women with a high acculturation (Turkish origin: aOR 1.62, 95%CI: 1.01–2.95 had a higher chance to deliver prematurely compared to women with a low acculturation (see model 3 in Table 2). The association between affinity to religion and preterm birth was not statistically significant. The regions of origin Latin America and Sub Saharan Africa showed statistically significant associations with preterm birth for all three models (Additional file 3, model 3, Latin American origin: aOR 2.17 95%CI: 1.03–5.57; model 3, Sub Saharan Africa origin: model 3, aOR 1.77, 95%CI: 1.05–2.99).

**Table 2:** Chance (expressed as Odds Ratios) to give birth prematurely, by country of origin, Berlin/Germany, 2011/12

		Model 1		Model 2		Model 3	
	n=	aOR (95% CI)	*p*-value	aOR (95% CI)	*p*-value	aOR (95% CI)	*p*-value
Country of origin							
Germany	2976	1.00		1.00		1.00	
Turkey	561	0.46 (0.32–0.68)	< 0.0001	0.46 (0.31–0.68)	< 0.0001	0.49 (0.33–0.73)	< 0.0001
Lebanon	317	0.70 (0.46–1.07)	0.098	0.71 (0.46–1.06)	0.097	0.71 (0.46–1.10)	0.114
Other countries	2559	0.82 (0.69–0.97)	0.051	0.82 (0.70–1.01)	0.049	0.87 (0.72–1.06)	0.168
Affinity to religion							
No religion	1777					1.00	
No affinity to religion	313					0.97 (0.65–1.44)	0.884
Low affinity to religion	664					0.90 (0.67–1.23)	0.515
Medium affinity to religion	1901					0.86 (0.68–1.09)	0.220
High affinity to religion	1758					1.01 (0.78–1.30)	0.969
Acculturation							
Low	387					1.00	
Medium	2016					1.49 (0.96–2.30)	0.074
High	4010					1.62 (1.01–2.59)	0.044
Education							
High	2521			1.00		1.00	
Medium	3071			0.91 (0.67–1.23)	0.546	0.97 (0.71–1.32)	0.821
Low	821			0.89 (0.72–1.09)	0.248	0.89 (0.71–1.32)	0.276
Age groups							
18–24 years	1288	1.00		1.00		1.00	
25–29 years	1732	0.96 (0.76–1.21)	0.726	0.96 (0.76–1.21)	0.726	0.96 (0.76–1.22)	0.735
30–34 years	1883	0.91 (0.72–1.15)	0.438	0.91 (0.72–1.15)	0.418	0.91 (0.72–1.15)	0.417
35+ years	1510	0.81 (0.63–1.04)	0.100	0.80 (0.63–1.04)	0.097	0.81 (0.63–1.04)	0.096
Family members in Berlin							
No	2114			1.00		1.00	
Yes	4299			1.08 (0.90–1.30)	0.420	1.07 (0.89–1.29)	0.481
Smoking							
No	5090	1.00		1.00		1.00	
Yes	1323	1.01 (0.82–1.24)	0.925	1.01 (0.81–1.26)	0.914	0.99 (0.80–1.24)	0.989
Household income (monthly)							
< 900 EUR	1572			1.00		1.00	
900–1500 EUR	1228			1.03 (0.81–1.32)	0.784	1.04 (0.81–1.32)	0.777
1500–2600 EUR	2122			0.91 (0.69–1.19)	0.472	0.90 (0.69–1.18)	0.446
> 2600 EUR	1491			0.92 (0.69–1.23)	0.577	0.92 (0.68–1.23)	0.564
Diabetes mellitus							
No	6353	1.00		1.00		1.00	
Yes	60	2.12 (1.11–4.03)	0.022	2.16 (1.13–4.11)	0.019	2.12 (1.11–4.04)	0.022
Preterm birth in anamneses							
No	6191	1.00		1.00			
Yes	222	1.78 (1.23–2.58)	0.002	1.81 (1.25–2.62)	0.002	1.82 (1.25–2.63)	0.002

13.7% of all newborns were SGA newborns. Women from Lebanon had the highest proportion; they delivered 20.8% SGA newborns (see Table 1). Table 3 shows the influence of the country of origin on SGA, controlling for medical variables, age and smoking (model 1), socioeconomic (model 2) variables, as well as affinity to religion and acculturation (model 3). In all three models the association between Lebanese origin and SGA was statistically significant (aOR: 1.72, 95%CI: 1.27–2.34) (see model 3 Table 3). The association between acculturation, affinity to religion, migration status (except Lebanese origin) and SGA was not statistically significant in any of the models.

**Table 3:** Chance (expressed as Odds Ratios) to give SGA birth, by country of origin, Berlin/Germany, 2011/12

		Model 1		Model 2		Model 3	
	n=	aOR (95% CI)	*p*-value	aOR (95% CI)	*p*-value	aOR (95% CI)	*p*-value
Country of origin							
Germany	2976	1.00		1.00		1.00	
Turkey	561	0.83 (0.62–1.10)	0.191	0.82 (0.62–1.10)	0.180	0.82 (0.62–1.10)	0.192
Lebanon	317	1.72 (1.28–2.30)	< 0.0001	1.72 (1.28–2.31)	< 0.0001	1.72 (1.27–2.34)	< 0.0001
Other countries	2559	1.03 (0.88–1.20)	0.742	1.02 (0.87–1.20)	0.781	1.03 (0.86–1.22)	0.762
Affinity to religion							
No religion	1777					1.00	
No affinity to religion	313					1.17 (0.84–1.63)	0.363
Low affinity to religion	664					0.91 (0.70–1.19)	0.503
Medium affinity to religion	1901					0.92 (0.75–1.13)	0.408
High affinity to religion	1758					0.96 (0.77–1.21)	0.737
Acculturation							
Low	387					1.00	
Medium	2016					1.09 (0.79–1.52)	0.592
High	4010					1.05 (0.73–1.51)	0.780
Education							
High	2521			1.00		1.00	
Medium	3071			0.86 (0.66–1.12)	0.263	0.89 (0.66–1.13)	0.300
Low	821			0.91 (0.76–1.08)	0.287	0.91 (0.79–1.09)	0.283
Age groups							
18–24 years	1288	1.00		1.00		1.00	
25–29 years	1732	0.98 (0.79–1.21)	0.866	0.98 (0.79–1.21)	0.859	0.98 (0.80–1.22)	0.870
30–34 years	1883	1.01 (0.81–1.23)	0.990	1.01 (0.82–1.24)	0.965	1.01 (0.82–1.24)	0.955
35+ years	1510	1.07 (0.86–1.33)	0.529	1.07 (0.86–1.33)	0.545	1.07 (0.86–1.33)	0.546
Family members in Berlin							
No	2114			1.00		1.00	
Yes	4299			0.93 (0.79–1.09)	0.361	0.93 (0.79–1.09)	0.376
Smoking							
No	5090	1.00		1.00		1.00	
Yes	1323	0.88 (0.73–1.05)	0.155	0.91 (0.75–1.10)	0.337	0.91 (0.75–1.10)	0.312
Household income (monthly)							
< 900 EUR	1572			1.00		1.00	
900–1500 EUR	1228			0.93 (0.75–1.14)	0.478	0.93 (0.76–1.15)	0.507
1500–2600 EUR	2122			0.97 (0.78–1.22)	0.816	0.98 (0.78–1.23)	0.845
> 2600 EUR	1491			0.93 (0.72–1.19)	0.550	0.94 (0.73–1.21)	0.615
Diabetes mellitus							
No	6353	1.00		1.00		1.00	
Yes	60	1.82 (0.98–3.38)	0.058	1.85 (0.99–3.45)	0.052	1.84 (0.99–3.42)	0.056

## Discussion

We found a Latina paradox for women of Turkish origin in Germany regarding preterm birth. The proportion of preterm births among Turkish women was half that of non-immigrant women and regression analyses confirmed that this protective effect persisted even after adjusting for a range of control variables. Our findings also confirm the hypothesis that some social factors are at play in explaining the Latina Paradox for Turkish women in Germany. A high acculturation level, i.e. a high affinity with the German culture and a less strong affinity with the country of origin, increases indeed the chance of preterm birth, erasing some of the protective effect that a Turkish origin may provide. In other words, low acculturation can be considered as a social factor which contributes to explaining the epidemiological paradox regarding preterm birth in our sample. There is no direct comparison in the literature. Several studies have used length of stay in the target country as a proxy variable for acculturation: immigrant women who had resided in Canada for fewer than 5 years had lower preterm risk than non-immigrant women, while those with ≥15 years of stay were at higher risk [38]. Latina women born abroad had better preterm birth outcomes than Latina women born in the US and non-Latina women [39], suggesting that the process of acculturation or integration to the new country attenuates the protective influence from which 1st generation immigrants benefit. More research is needed to identify which specific items of acculturation are responsible for the differences in preterm birth rates.

We also explored the role of affinity to religion as another social factor. First generation women more often reported a medium or high affinity to religion compared to non-immigrant and 2nd generation women. However, regression analyses showed no statistically significant association between affinity to religion and preterm birth. We do not find the protective effect of religiosity on perinatal outcomes that has been shown for Latin American women in the US, where, for example, specific religious groups may promote positive health behaviours during pregnancy (e.g. no smoking, no alcohol) [3]. When trying to test the hypothesis that other religions may have a positive effect on perinatal outcomes through social support in the community, [3] we found no evidence indicating that high affinity to religion tends to lead to favourable health behaviour during pregnancy.

Still with regard to preterm birth, we found that women from Latin America and Sub Saharan Africa have higher odds of having a preterm birth than German women. Although a study which investigated factors for preterm birth in Germany found no significant association between a Latin American or Sub Saharan African origin and the outcome [40], evidence from other countries has shown on different occasions that immigrant women from Sub Saharan Africa tend to have poorer birth outcomes than the native population (see for example [41]). We found no equivalent in the European literature of the relatively poor birth outcomes of Latin American women in our cohort.

In line with some of the existing literature, we found no epidemiological paradox with regard to SGA [38, 39]. On the contrary, descriptive statistics showed that SGA was more frequent among 1st generation women compared to non-immigrant women, and that 1st generation immigrants from Lebanon had SGA rates about 1.5 times those of non-immigrant women. This resonates with evidence from the US where women of a Middle Eastern origin were found to have higher SGA rates than non-immigrant women [5]. After adjusting for socioeconomic status, acculturation and affinity to religion, Lebanon as a country of origin remained the only significant factor influencing SGA rates. Higher SGA rates in women of Lebanese origin may therefore at least partly be explained by non-social factors; one hypothesis being that standard SGA calculation based on birth-weight tables for Germans may not be an adequate measure for newborns from other ethnicities, with different average body types [14].

### Strengths and limitations

Data were collected in a highly-standardised way in a large sample of women with a high response rate, comprising information on migrant status and other socioeconomic parameters which are lacking in routine perinatal data. A limitation is the restriction to Berlin where the proportion of immigrants in the population is high. Hence, the results cannot be generalised to rural areas or smaller cities where social support for immigrant women may be smaller. The absolute numbers of immigrant women from countries other than Turkey and Lebanon were too small for stratified analyses. Diabetes mellitus recorded on the antenatal card was used for analyses. This may lead to an underestimation of the gestational diabetes prevalence. Acculturation is difficult to measure and may be conflated with specific social determinants of health [42]. However, this may not be the case here, given that we could demonstrate differing effects for preterm birth and SGA.

We excluded nearly 21% from all deliveries in the hospitals (gross sample size). 9.2% of the women did not consent or could not be reached. 3.8% of the women were excluded based on the inclusion and exclusion criteria [17] and 8% of the women had missing values. The excluded women had a lower monthly household income and a lower education compared to the included women. This could possibly form a selection bias. As we found no difference between included and excluded women regarding preterm birth and SGA and the proportion (as well as the number) of included women is high we assume that the named differences have probably no influence on our conclusion. Nevertheless readers should consider the selection bias while reading our conclusion.

## Conclusion

There is evidence that a social factor, namely the level of acculturation of pregnant women with a migration background, contributes to explaining the epidemiological paradox regarding preterm birth. Furthermore, independently of social factors, Turkey as country of origin has a protective influence on preterm birth whereas Lebanon as country of origin has a negative influence on SGA. Hence, the direction of the observed effect varies depending on the country and the outcome. This suggests that non-social factors that relate to the country of origin contribute to explaining differences in birth outcomes. Service providers therefore need to take a more differentiated view of the potential risks immigrant women face than what might be implied by a broad term like “Latina paradox” or “healthy migrant effect”.

## Additional files

Additional file 1: Distribution of socio-demographic characteristics and obstetric indicators among 1st generation immigrant women, Berlin, Germany, 2011/12. Additional information about 1st immigration women. (DOCX 20 kb)

Additional file 2: Chi Square Test for premature birth, by region of origin, Berlin/Germany, 2011/12. Results from Chi Square Test for premature birth, by country of origin (DOCX 13 kb)

Additional file 3: Chance (expressed as Odds Ratios) to give birth prematurely, by region of origin, Berlin/Germany, 2011/12. Additional regression analyses for Latin America & Caribbean and Sub Saharan Africa (countries which had significant associations with preterm birth in Chi Square Tests) (DOCX 16 kb)

## Abbreviations

aOR: Adjusted odds ratio
CI: Confidence intervals
LBW: Low birth weights
OR: Odds ratio
SD: Standard deviation
SGA: Small-for-gestational-age
