# Supplementary material for: Do social factors and country of origin contribute towards explaining a “Latina paradox” among immigrant women giving birth in Germany?
Source: BMC Public Health. 2019 Feb 12;19:181. doi: 10.1186/s12889-019-6523-9 (PMC6373125; doi:10.1186/s12889-019-6523-9)
Supplement: Supplementary file 1 — Distribution of socio-demographic characteristics and obstetric indicators among 1st generation immigrant women, Berlin, Germany, 2011/12. Additional information about 1st immigration women. (DOCX 20 kb) [file 12889_2019_6523_MOESM1_ESM.docx]

Additional file 1 Distribution of socio-demographic characteristics and obstetric indicators among 1^st^ generation immigrant women, Berlin, Germany, 2011/12

|  | |  | |  | |  |  |  |  |  |  |  |  |  |  |  |  |
| --- | --- | --- | --- | --- | --- | --- | --- | --- | --- | --- | --- | --- | --- | --- | --- | --- | --- |
|  | | | total  (all origins) | | EU15 | | EU27 | Other Europe | Middle East * | Lebanon | Turkey | North Africa | Sub-Saharan Africa | Far East | Latin America & Caribbean | North America | Oceania |
| **Study population (n)** | | | 2,552 | | 127 | | 357 | 369 | 133 | 317 | 561 | 67 | 85 | 127 | 44 | 360 | 5 |
| **Maternal age** | **n=** | |  | |  | |  |  |  |  |  |  |  |  |  |  |  |
| 18-24 years | 509 | | 19.9 | | 19.7 | | 24.1 | 19.0 | 19.0 | 19.6 | 20.0 | 25.4 | 21.2 | 19.7 | 6.8 | 17.2 | 20.0 |
| 25-29 years | 696 | | 23.7 | | 22.8 | | 24.1 | 30.4 | 24.8 | 26.2 | 26.4 | 23.9 | 25.9 | 25.2 | 25.0 | 26.9 | 40.0 |
| 30-34 years | 752 | | 29.5 | | 33.9 | | 26.9 | 28.7 | 27.6 | 27.4 | 28.5 | 35.8 | 23.5 | 30.7 | 38.6 | 31.9 | 20.0 |
| 35+ years | 595 | | 23.3 | | 23.6 | | 24.9 | 22.0 | 28.6 | 26.8 | 25.1 | 24.9 | 29.4 | 24.4 | 29.5 | 23.9 | 20.0 |
| **Educational attainment** | **n=** | |  | |  | |  |  |  |  |  |  |  |  |  |  |  |
| High | 877 | | 34.4 | | 26.8 | | 35.3 | 35.5 | 30.5 | 39.4 | 41.0 | 32.8 | 34.1 | 33.9 | 38.6 | 42.5 | 20.0 |
| Medium | 1,035 | | 40.6 | | 44.9 | | 37.5 | 38.5 | 31.4 | 47.0 | 45.8 | 50.7 | 43.5 | 42.5 | 38.6 | 42.8 | 60.0 |
| Low | 640 | | 25.1 | | 28.3 | | 27.2 | 26.0 | 38.1 | 13.6 | 13.2 | 16.4 | 22.4 | 23.6 | 22.7 | 14.7 | 20.0 |
| **Family members in**  **Berlin** | **n=** | |  | |  | |  |  |  |  |  |  |  |  |  |  |  |
| Yes | 1,353 | | 53.0 | | 58.3 | | 52.9 | 44.4 | 53.3 | 61.5 | 67.6 | 47.8 | 49.4 | 52.0 | 47.7 | 54.4 | 60.0 |
| No | 1,199 | | 47.0 | | 41.7 | | 47.1 | 55.6 | 46.7 | 38.5 | 32.4 | 52.2 | 50.6 | 48.0 | 52.3 | 45.6 | 40.0 |
| **Affinity to religion** | **n=** | |  | |  | |  |  |  |  |  |  |  |  |  |  |  |
| No religion | 254 | | 9.1 | | 11.8 | | 10.1 | 8.7 | 12.4 | 8.1 | 8.8 | 9.0 | 11.8 | 5.5 | 20.5 | 11.7 | 0.0 |
| No affinity to religion | 89 | | 3.0 | | 0.8 | | 3.1 | 3.8 | 2.9 | 1.9 | 3.5 | 4.5 | 0.0 | 3.1 | 4.5 | 5.8 | 0.0 |
| Low affinity to religion | 214 | | 8.6 | | 9.4 | | 8.4 | 9.8 | 8.6 | 6.3 | 8.2 | 3.0 | 8.2 | 5.5 | 13.6 | 8.1 | 0.0 |
| Medium affinity to religion | 947 | | 37.2 | | 34.6 | | 38.9 | 38.2 | 32.4 | 30.6 | 36.6 | 53.7 | 36.5 | 37.0 | 29.5 | 36.1 | 40.0 |
| High affinity to religion | 1,048 | | 42.1 | | 43.3 | | 39.5 | 39.6 | 43.8 | 53.1 | 42.9 | 29.9 | 43.5 | 48.8 | 31.8 | 31.8 | 60.0 |
| **Smoking during pregnancy** | **n=** | |  | |  | |  |  |  |  |  |  |  |  |  |  |  |
| No | 2,060 | | 80.7 | | 73.2 | | 79.6 | 77.8 | 81.9 | 80.1 | 77.9 | 80.6 | 80.0 | 77.2 | 86.4 | 84.4 | 80.0 |
| Yes | 492 | | 19.3 | | 26.8 | | 20.4 | 22.2 | 18.1 | 19.9 | 22.1 | 19.4 | 20.0 | 22.8 | 13.6 | 15.6 | 20.0 |
| **Household income (monthly)** | **n=** | |  | |  | |  |  |  |  |  |  |  |  |  |  |  |
| <900 EUR | 505 | | 19.5 | | 7.1 | | 6.7 | 8.9 | 3.9 | 13.8 | 20.8 | 6.0 | 18.8 | 7.9 | 4.5 | 44.2 | 0.0 |
| 900-1500 EUR | 692 | | 26.5 | | 22.8 | | 29.4 | 26.0 | 21.9 | 34.4 | 29.3 | 43.3 | 38.8 | 45.7 | 4.5 | 25.6 | 0.0 |
| 1500-2600 EUR | 1,070 | | 42.9 | | 63.8 | | 53.5 | 51.5 | 50.4 | 38.1 | 37.2 | 46.3 | 28.2 | 39.4 | 54.5 | 20.3 | 100.0 |
| >2600 EUR | 285 | | 11.1 | | 6.3 | | 10.4 | 13.6 | 23.8 | 13.7 | 12.6 | 4.5 | 14.2 | 7.1 | 36.4 | 9.9 | 0.0 |
| **Diabetes mellitus** | **n=** | |  | |  | |  |  |  |  |  |  |  |  |  |  |  |
| no | 2,536 | | 99.4 | | 99.2 | | 99.2 | 99.4 | 99.8 | 99.7 | 99.6 | 100.0 | 100.0 | 99.8 | 99.4 | 99.1 | 100.0 |
| yes | 16 | | 0.6 | | 0.8 | | 0.8 | 0.6 | 0.2 | 0.3 | 0.4 | 0.0 | 0 | 0.2 | 0.6 | 0.9 | 0.0 |
| **Preterm birth in anamneses** | **n=** | |  | |  | |  |  |  |  |  |  |  |  |  |  |  |
| no | 2,471 | | 96.8 | | 97.6 | | 96.9 | 97.0 | 94.3 | 95.3 | 97.9 | 95.5 | 96.5 | 96.1 | 97.7 | 96.7 | 100.0 |
| yes | 81 | | 3.2 | | 2.4 | | 3.1 | 3.0 | 5.7 | 4.7 | 2.1 | 4.5 | 3.5 | 3.9 | 2.3 | 3.3 | 0.0 |
| **Acculturation** | **n=** | |  | |  | |  |  |  |  |  |  |  |  |  |  |  |
| Low | 364 | | 13.9 | | 14.2 | | 14.0 | 16.0 | 16.2 | 10.0 | 6.1 | 9.0 | 18.9 | 14.2 | 13.6 | 10.3 | 0.0 |
| Medium | 1,035 | | 59.3 | | 61.4 | | 62.2 | 59.3 | 59.0 | 61.9 | 61.2 | 53.7 | 57.6 | 62.2 | 59.1 | 52.5 | 60.0 |
| High | 877 | | 26.8 | | 24.4 | | 23.8 | 24.7 | 24.8 | 28.1 | 32.7 | 37.3 | 23.5 | 23.6 | 27.3 | 37.2 | 40.0 |
| **Preterm birth** | **n=** | |  | |  | |  |  |  |  |  |  |  |  |  |  |  |
| No | 2,318 | | 90.7 | | 94.5 | | 89.4 | 89.4 | 91.4 | 91.8 | 94.7 | 91.0 | 88.2 | 94.5 | 88.6 | 91.4 | 80.0 |
| Yes | 234 | | 9.3 | | 5.5 | | 10.6 | 10.6 | 8.6 | 8.2 | 5.3 | 9.0 | 11.8 | 5.5 | 11.4 | 8.6 | 20.0 |
| **SGA birth** | **n=** | |  | |  | |  |  |  |  |  |  |  |  |  |  |  |
| No | 2,187 | | 85.7 | | 83.5 | | 83.4 | 85.4 | 82.9 | 79.2 | 88.8 | 86.6 | 88.2 | 88.2 | 90.9 | 85.6 | 60.0 |
| Yes | 365 | | 14.3 | | 16.5 | | 16.6 | 14.6 | 17.1 | 20.8 | 11.2 | 13.4 | 11.8 | 11.8 | 9.1 | 14.4 | 40.0 |

*=excluding Turkey and Lebanon
