# Supplementary material for: Do social factors and country of origin contribute towards explaining a “Latina paradox” among immigrant women giving birth in Germany?
Source: BMC Public Health. 2019 Feb 12;19:181. doi: 10.1186/s12889-019-6523-9 (PMC6373125; doi:10.1186/s12889-019-6523-9)
Supplement: Supplementary file 2 — Chi Square Test for premature birth, by region of origin, Berlin/Germany, 2011/12. Results from Chi Square Test for premature birth, by country of origin (DOCX 13 kb) [file 12889_2019_6523_MOESM2_ESM.docx]

**Additional file 2** Chi Square Test for premature birth, by region of origin, Berlin/Germany, 2011/12

|  | | Preterm birth | | SGA | |  |
| --- | --- | --- | --- | --- | --- | --- |
| n= | | p-value | Phi-coefficient | | p-value | Phi-coefficient |
| **Country of origin** |  |  |  | |  |  |
| EU15 | 127 | 0.065 | -0.023 | | 0.455 | -0.009 |
| EU27 | 357 | 0.688 | 0.005 | | 0.536 | 0.008 |
| Other Europe | 369 | 0.186 | -0.016 | | 0.803 | 0.003 |
| Middle East (excluding Lebanon and Turkey) | 133 | 0.828 | -0.004 | | 0.396 | 0.017 |
| North Africa | 67 | 0.097 | -0.021 | | 0.243 | -0.015 |
| Sub-Saharan Africa | 85 | 0.009 | 0.033 | | 0.523 | -0.008 |
| Far East | 127 | 0.401 | -0.010 | | 0.230 | -0.015 |
| Latin America & Caribbean | 44 | 0.020 | 0.029 | | 0.348 | -0.012 |
| North America | 360 | 0.634 | -0.006 | | 0.891 | -0.002 |
| Oceania | 5 | - | - | | - | - |
| Germany | 2,976 | <0.0001 | 0.045 | | 0.594 | -0.007 |
| Turkey | 561 | <0.0001 | -0.046 | | 0.078 | -0.022 |
| Lebanon | 317 | 0.333 | -0.12 | | <0.0001 | 0.047 |
