# Supplementary material for: Do social factors and country of origin contribute towards explaining a “Latina paradox” among immigrant women giving birth in Germany?
Source: BMC Public Health. 2019 Feb 12;19:181. doi: 10.1186/s12889-019-6523-9 (PMC6373125; doi:10.1186/s12889-019-6523-9)
Supplement: Supplementary file 3 — Chance (expressed as Odds Ratios) to give birth prematurely, by region of origin, Berlin/Germany, 2011/12. Additional regression analyses for Latin America & Caribbean and Sub Saharan Africa (countries which had significant associations with preterm birth in Chi Square Tests) (DOCX 16 kb) [file 12889_2019_6523_MOESM3_ESM.docx]

| **Additional file 3** Chance (expressed as Odds Ratios) to give birth prematurely, by region of origin, Berlin/Germany, 2011/12 | | | | | | | | |
| --- | --- | --- | --- | --- | --- | --- | --- | --- |
|  | | Model 1 | | Model 2 | |  | Model 3 |  |
| n= | | aOR (95% CI) | p-value | | aOR (95% CI) | p-value | aOR (95% CI) | p-value |
| **Region of origin** |  |  |  | |  |  |  |  |
| Germany | 2976 | 1.00 |  | | 1.00 |  | 1.00 |  |
| Latin America & Caribbean | 44 | 2.14 (1.02-4.47) | 0.044 | | 2.13 (1.02-3.96) | 0.045 | 2.17 (1.03-4.57) | 0.040 |
| Sub Saharan Africa | 85 | 1.73 (1.03-2.92) | 0.038 | | 1.75 (1.04-2.96) | 0.035 | 1.77 (1.05-2.99) | 0.033 |
| Other countries | 3308 | 0.72 (0.57-0.97) | 0.046 | | 0.89 (0.76-1.01) | 0.096 | 0.90 (0.89-1.18) | 0.275 |
| **Affinity to religion** |  |  |  | |  |  |  |  |
| No religion | 1777 |  |  | |  |  | 1.00 |  |
| No affinity to religion | 313 |  |  | |  |  | 0.98 (0.66-1.46) | 0.926 |
| Low affinity to religion | 664 |  |  | |  |  | 0.88 (0.65-1.12) | 0.420 |
| Medium affinity to religion | 1901 |  |  | |  |  | 0.84 (0.67-1.06) | 0.138 |
| High affinity to religion | 1758 |  |  | |  |  | 0.98 (0.76-1.27) | 0.897 |
| **Acculturation** |  |  |  | |  |  |  |  |
| Low | 387 |  |  | |  |  | 1.00 |  |
| Medium | 2016 |  |  | |  |  | 1.52 (0.98-2.31) | 0.062 |
| High | 4010 |  |  | |  |  | 1.62 (1.11-2.73) | 0.016 |
|  |  |  |  | |  |  |  |  |
| **Education** |  |  |  | |  |  |  |  |
| High | 2521 |  |  | | 1.00 |  | 1.00 |  |
| Medium | 3071 |  |  | | 0.86 (0.64-1.16) | 0.312 | 0.96 (0.71-1.32) | 0.815 |
| Low | 821 |  |  | | 0.87 (0.71-1.07) | 0.193 | 0.89 (0.72-1.09) | 0.257 |
|  |  |  |  | |  |  |  |  |
| **Age groups** |  |  |  | |  |  |  |  |
| 18 – 24 years | 1288 | 1.00 |  | | 1.00 |  | 1.00 |  |
| 25 – 29 years | 1732 | 0.96 (0.76-1.21) | 0.716 | | 0.96 (0.75-1.22) | 0.722 | 0.96 (0.76-1.22) | 0.741 |
| 30 – 34 years | 1883 | 0.91 (0.72-1.15) | 0.405 | | 0.90 (0.71-1.14) | 0.394 | 0.90 (0.71-1.14) | 0.397 |
| 35+ years | 1510 | 0.81 (0.63-1.04) | 0.096 | | 0.81 (0.63-1.04) | 0.094 | 0.81 (0.63-1.04) | 0.099 |
| **Family members in Berlin** |  |  |  | |  |  |  |  |
| No | 2114 |  |  | | 1.00 |  | 1.00 |  |
| Yes | 4299 |  |  | | 1.1 (0.91-1.32) | 0.322 | 1.07 (0.88-1.28) | 0.508 |
| **Smoking** |  |  |  | |  |  |  |  |
| No | 5090 | 1.00 |  | | 1.00 |  | 1.00 |  |
| Yes | 1323 | 1.01 (0.82-1.24) | 0.944 | | 1.03 (0.80-1.27) | 0.787 | 0.99 (0.80-1.24) | 0.995 |
| **Household income (monthly)** |  |  |  | |  |  |  |  |
| < 900 EUR | 1572 |  |  | | 1.00 |  | 1.00 |  |
| 900-1500 EUR | 1228 |  |  | | 1.04 (0.81-1.32) | 0.775 | 1.04 (0.81-1.32) | 0.776 |
| 1500-2600 EUR | 2122 |  |  | | 0.92 (0.71-1.20) | 0.547 | 0.89 (0.68-1.16) | 0.379 |
| >2600 EUR | 1491 |  |  | | 0.97 (0.73-1.23) | 0.829 | 0.91 (0.68-1.22) | 0.513 |
|  |  |  |  | |  |  |  |  |
| **Diabetes mellitus** |  |  |  | |  |  |  |  |
| No | 6353 | 1.00 |  | | 1.00 |  | 1.00 |  |
| Yes | 60 | 2.14 (1.12-4.09) | 0.021 | | 2.19 (1.14-4.18) | 0.018 | 2.12 (1.11-4.06) | 0.023 |
|  |  |  |  | |  |  |  |  |
| **Preterm birth in anamneses** |  |  |  | |  |  |  |  |
| No | 6191 | 1.00 |  | | 1.00 |  |  |  |
| Yes | 222 | 1.86 (1.29-2.70) | 0.001 | | 1.89 (1.30-2.70) | 0.001 | 2.12 (1.31-2.75) | 0.001 |
